# Spanish society of laboratory medicine external quality assurance programmes: evolution of the analytical performance of clinical laboratories over 30 years and comparison with other programmes

**DOI:** 10.1515/almed-2020-0019

**Published:** 2020-05-19

**Authors:** Carmen Perich, Carmen Ricós, Fernando Marqués, Joana Minchinela, Angel Salas, Cecilia Martínez-Bru, Beatriz Boned, Rubén Gómez-Rioja, Marià Cortés, Elisabet González-Lao, Jose Vicente García Lario, Xavier Tejedor, Sandra Bullich, Montserrat Ventura, Ricardo González-Tarancón, Pilar Fernández-Fernández, Francisco Ramón, Zoraida Corte, Antonia Ma Llopis, Jorge Díaz-Garzón, Margarita Simón, Pilar Fernández-Calle

**Affiliations:** Comité de Programas Externos de la SEQC-ML, Barcelona, Spain; Comisión de Calidad Analítica de la SEQC-ML, Barcelona, Spain

**Keywords:** analytical performance specifications, biological variation, external quality assurance programmes, harmonisation, state of the art

## Abstract

The purpose of this study is to understand the evolution of the analytical performance of the laboratories participating in the Spanish society of laboratory medicine (SEQC^ML^) external quality assurance (EQA) programmes during its 30 years of operation and to compare it with the performance of other EQA programmes to establish whether the results are similar. The results obtained during this period are evaluated by applying the biological variability (BV) and state of the art-derived quality specifications. In addition, the results are compared with those obtained by other EQA programme organisations. It is noted that the laboratories participating in the EQA–SEQC^ML^ programmes have improved their performance over 30 years of experience and that the specifications derived from biological variation are achievable. It is difficult to compare EQA programmes, due to lack of accessibility and the differences in the design of these programmes (control materials, calculations used and analytical specifications established). The data from this study show that for some biological magnitudes the results obtained by the programmes are not yet harmonised, although efforts are being made to achieve this. Organisers of EQA programmes should also join the harmonisation effort by providing information on their results to enable comparison.

## Introduction

External quality assurance (EQA) programmes are a key component of laboratory quality management. Their involvement enables the laboratory to evaluate and monitor its analytical performance, compare the reliability of measurement procedures and understand the degree of harmonisation of analytical results.

The Spanish Society of Laboratory Medicine (SEQC^ML^) launched its first EQA programme in 1981, including 20 biochemical serum magnitudes and with 147 participants [1]. Since then, we have incorporated our own programmes and established collaborations with national and European programmes (Spanish Society of Haematology and Haemotherapy for haematological magnitudes, UK NEQAS for immunology and allergy, and OELM for trace elements). The SEQC^ML^ currently distributes 29 programmes, for all laboratory areas, covering 189 biological magnitudes and involving around 700 laboratories.

The information to be provided by an EQA programme according to the *Task Group on Performance Specifications for EQAS* of the European Federation of Clinical Chemistry and Laboratory Medicine (EFLM-TG-EQA) created at the 1st Strategic Conference in Milan in 2014 [2], in order to allow participants to correctly interpret their results [3] involves: the control material distributed, the target value to which the laboratory result is compared, how the analytical performance of the participant is expressed and the evaluation criterion used.

The control material for most SEQC^ML^ EQA programmes is stabilised serum except for the commutable serum programme (SCR) with reference method values assignment (SCR) which uses frozen human serum.

The target value of the control samples is the average value obtained by each group of users of the same analytical method and instrument (homogeneous group or peer group), and that of the SCR programme is the certified reference value.

The analytical performance of the laboratory is expressed by the percentage deviation (PD%) of each result from target value, indicative of total analytical error (ET). The analytical performance of the participating methods is expressed by the coefficient of variation (CV) between laboratories, which indicates the imprecision of each homogeneous group, and by the PD% of the average for each homogeneous group, indicating the bias of that method.

The assessment criterion has changed over the years: until 1993 the statistical criterion of the standard score with respect to the homogeneous group was used [4]. From 1994 onwards, the analytical quality specification (AQS) derived from the biological variability (BV) was also applied, where data were available [5] and otherwise, after 2011, the 90th percentile of the results of the individual deviations (PD%) obtained by the participants, after exclusion of extreme values, was applied.

In the case of using BV, three “levels” of demand are used, called minimum, desirable and optimum [6], according to the percentage of individual deviations of the laboratories complying with the specification:

– Minimum: less than 80% of PD%s would comply
– Desirable: between 80 and 90%
– Optimal: 90% or more PD% would be within the limit defined by BV.

Following the categorisation of EQA programmes described by Miller et al. [7] most of the programmes in the SEQC^ML^ are category 4 (control material for stabilised human serum, value assigned by homogeneous group, repeated measurements, EPA based on BV); the SCR programme is category 1 (control material for frozen human serum, value assigned by reference method, repeated measurements, EPA based on BV).

The objectives of this study are to understand the evolution of the analytical performance of the laboratories participating in the SEQC^ML^ EQA programmes over more than 30 years of operation (1981 – 2018), and to compare the analytical performance obtained by laboratories participating in the SEQC^ML^ EQA programmes with that of participants in other EQA programmes.

## Materials and methods

The material used in this study is the reports for the individual and end-of-cycle laboratory, which the SEQC^ML^ EQA programmes provide to the participants.

### Report for the individual laboratory

This has evolved over time and now meets the recommendations of EFLM-TF-EQA [3]. The data included are:

– Histogram of distribution of results.
– Number of results received.
– Number of results included in the statistical calculations (aberrant values, which repeatedly exceed the mean interval ± 3 standard deviations, are excluded until no data fall outside this interval) [8].
– Mean and standard deviation of all participants, of the same method as the participant and of the same method and instrument (homogeneous group).
– Result obtained by the laboratory and its deviation from the target value, expressed as a standard score and as a percentage.
– Acceptable percentage deviation based on the criterion derived from BV for analytical ET, a graph with the results of the previous 12 months and their deviation from the BV (Supplementary Figure 1) having been added in 2005.

Under the SCR programme the report includes:

– Percentage deviation from the reference value for the 6 samples,
– CV (obtained between control replicates) for the laboratory,
– Mean value, CV and percentage deviation mean of the homogeneous group (same method, instrument and traceability) from the reference value (A of Supplementary Figure 2)

In line with its educational purpose, the organisation prepares and sends technical notes relating to the programmes in order to help participants interpret the results. There is also two-way communication for any queries that participating laboratories may have.

### End-of-cycle report

This report contains the indicators for the analytical performance of the participating methods:

– Inter-laboratory variation coefficient within homogeneous groups.
– Homogeneous group bias (difference between group mean and target value).
– Since 2011, percentages of the deviation of each result in comparison to the homogeneous group (PD%). Initially the 90th percentile (P90) was shown, but from 2017 onwards P20, P50 and P70 are also included so that each laboratory can select its specification.

The method used is visual inspection of the end-of-cycle reports issued from 1981 to 2018, quantification of the percentage of results in compliance with the EPA and a visual comparison of the indicators with those of programmes in other countries.

## Results

### Change in the analytical performance of laboratories participating in the SEQC^ML^ EQA programmes

**Figure 1:**
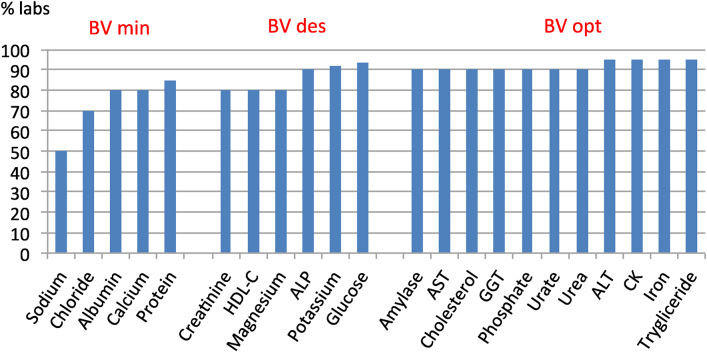
Compliance with the BV-derived total analytical error specification of the 2018 Serum Biochemistry Programme.

The individual report provides the deviation from the peer group. Figure 1 shows the percentage of individual laboratory results (taken from individual reports) in 2018, whose deviation (PD%) from the homogeneous group meets the quality specification for analytical ET derived from the BV (minimum, desirable or optimal), on the serum biochemistry programme.

For most biological quantities, 90% of the participants' results meet the specification and even for quantities with higher homoeostatic regulation (lower BV and more restrictive specifications), 50% or more of laboratories are already able to meet the requirement.

A comparison of the 90th percentile PD% for the laboratories participating in the hormone programme between 2012 and 2018, a very important decrease can be observed (Supplementary Figure 3). 90% of laboratory insulin results in 2012 had a PD = 39%, which decreased to a PD% = 14% in 2018. Likewise, the P90 of the C-peptide results decreased from 23 to 14% and the thyrotropin results from 13 to 9%.

**Figure 2:**
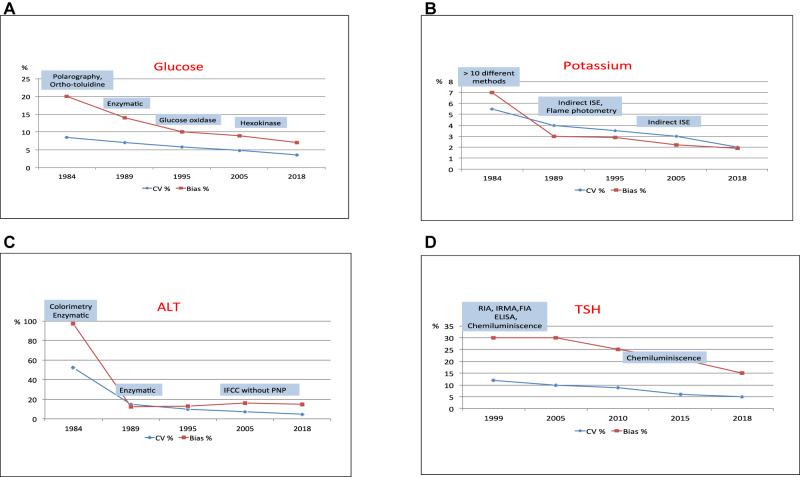
Evolution of inter-laboratory imprecision and bias towards the homogeneous group.

The end-of-cycle report shows the indicators of inter-laboratory imprecision (measured as CV) and bias towards the homogeneous group (measured as PD%). Figure 2 shows five time slices of magnitudes chosen as representative of substrates (glucose), electrolytes (potassium), Alanine aminotransferase (ALT) and thyroid-stimulating hormone (TSH):

– Glucose (Figure 2A): the predominant use of enzymatic methods since the 1990s reduced inter-laboratory imprecision to 6% and bias to 10%. With the use of more specific enzymatic methods, a CV of 4% and a PD% of 7% were obtained in 2018.
– Potassium (Figure 2B): in 1984 there were more than 10 possible methods for analysing potassium, with a CV of 5.5% and a bias of 7%; from 1989 to 2000 there were flame photometry and potentiometry together and subsequently only potentiometry. Analytical performance indicators gradually improved to a CV of 2% and a bias of 2% by 2018.
– ALT (Figure 2C): as with other enzymes, the disappearance of colourimetric methods led to a reduction in inter-laboratory imprecision, from CVs close to 50% to CVs below 5%. Bias also decreased dramatically.
– TSH (Figure 2D): the hormone programme was started in 1999, using different analytical methods with a CV of 12% and a bias of 30%. As of 2010, with the predominant use of luminescent methods, the CV had decreased to 5% and the bias to 15%.

### Comparison of SEQC^ML^ EQA programmes with other EQA programmes

Table 1 shows the inter-laboratory imprecision obtained in 2018 by the SEQC^ML^ (non-commutable control) programme [8], the Dutch Stichting Kwaliteitsbewaking *Medische Laboratoriumdiagnostiek (SKML*) programme (commutable control) [9], the Belgian Empower *project* (commutable control) [10] and the German Referenzinstitutfür *Bioanalytik (non-commutable control*) [11], for some biological parameters in serum biochemistry. In general the CV are similar across the different programmes, except for ALT (CV between 4 and 10%) and LDH (CV between 2.6 and 9.0%).

**Table 1:** Inter-laboratory imprecision (CV%) obtained by several European programmes in 2018.

Magnitude	SEQC (NC) [8]	SKML (C) [9]	*Empower project* (C) [10]	*RfB* (NC) [11]
Albumin	4.5	4.0	–	4.7
ALT	4.9	10.0	9.1	5.5
AST	5.0	7.5	3.3	5.7
Calcium	2.3	2.0	–	2.7
Creatinine	5.7	5.5	–	4.4
Phosphate	3.3	4.0	–	3.6
GGT	5.7	8.5	6.9	4.8
Glucose	3.4	4.0	–	3.4
LDH	5.1	9.0	2.6	4.4
Magnesium	3.4	6.0	–	4.0
Potassium	2.0	1.0	1.2	2.3
Sodium	1.6	0.9	0.8	1.9
Urate	2.6	3.0	–	4.0
Urea	3.5	4.0	–	4.8

C, commutable samples; NC, non-commutable samples.

Table 2 visually compares the PD% to the peer group obtained in the SEQC^ML^ and ProBioQual (France) programmes [12]; both use non-commutable material and the peer group as the target value. For this indicator, better results are observed in the SEQCML programme (visually lower PD%).

**Table 2:** Total analytical error (PD% compared to peer group) obtained by the 90th percentile of participants in two European programmes.

Magnitude	SEQC^ML^ [8] 2018	PBQ [12] 2015
Alkaline Phosphatase	10.4	15.1
ALT	7.5	11.7
Amylase	7.2	9.1
AST	7.8	10.8
Bilirubin	8.4	12.0
Calcium	4.3	5.7
Cholesterol	5.5	6.6
Creatine kinase	8.3	11.8
Chlorine	3.9	5.6
Creatinine	9.2	11.0
GGT	9.7	14.1
Glucose	5.0	7.3
Potassium	3.2	4.6
LDH	9.9	15.2
Lipase	11.0	13.3
Magnesium	6.0	10.1
Sodium	2.7	3.6
Phosphate	5.9	8.2
Triglyceride	6.5	8.6
Urate	5.4	5.9
Urea	6.4	12.5

Non-commutable controls. Target value: average of the homogeneous group.

Table 3 presents the 2018 results of the bias (PD%) obtained in three programmes using commutable controls with values assigned by certified reference methods (SEQC^ML^–SCR, SKML and Empower *Project) (8,10,*13). The values shown correspond to the percentage deviation between the average of the most frequent peer group and the reference value. The data are for enzymes determined by methods recommended by IFCC. A lower bias (visually lower PD%) is observed in the results of the SEQC^ML^ and SKML programmes.

**Table 3:** Bias (PD%) with respect to the reference method obtained for enzymes determined by the IFCC-recommended methods (2018).

Magnitude	SEQC [8]	SKML [10]	Empower Project [13]
Alkaline Phosphatase	−6.0	−10.0	−15.0
ALT	−16.0	−14.0	−25.0
AST	−12.0	−11.0	−26.0
Creatine kinase	3.0	4.0	
GGT	−5.5	−8.0	−15.0
LDH	−8.0	−5.0	−18.0

Table 4 shows the observations published in different studies [8], [13], [14], [15], [16] regarding the comparability of results between method-instrument systems, using commutable control materials. The authors consider that there is good harmonisation for urea, cholesterol, glucose, uric acid and potassium and poor standardisation for creatinine, calcium, magnesium, chlorine and sodium; while results for protein and phosphate are discrepant. However, it is important to emphasise that these organisations used different EPAs (state of the art, BV), to consider the degree of harmonisation of these biological magnitudes.

**Table 4:** Degree of comparability between method-instrument systems according to several studies.

Magnitude	SCR SEQC [8]	SKML [13]	Empower [14]	Miller [15]	Van Houcke [16]
Urea	–	YES	–	YES	–
Cholesterol	–	YES	YES	YES	–
Glucose	DEP	YES	YES	YES	–
Creatinine	NO	NO	DEP	–	–
Phosphate	–	–	YES	DEP	–
Protein	DEP	DEP	YES	–	YES
Urate	YES	YES	YES	YES	–
Calcium	DEP	NO	NO	–	DEP
Magnesium	DEP	DEP	DEP	DEP	DEP
Chlorine	DEP	NO	DEP	NO	–
Potassium	YES	YES	YES	YES	–
Sodium	NO	NO	YES	NO	–

YES, standardised method-instrument systems; NO, non-standardised method-instrument systems; DEP, some systems are standardised and others are not.

## Discussion

The control material for an EQA should be similar to patient samples and the target value should be determined by certified reference methods [3]. Only one of the 29 SEQC programmes currently meets these conditions (SCR), the main difficulties being a lack of commutable control materials and reference methods for all biological quantities in the clinical laboratory.

If the EQA programme uses commutable material with a value assigned by reference methods, it can evaluate the true accuracy of the individual laboratory, assess analytical methods and estimate bias and promote standardisation. Otherwise, it can only assess the individual laboratory and compare homogeneous groups.

Indicators of analytical performance: The analytical ET, calculated imprecision and bias, and type of EPA used in the SEQC^ML^ programmes to evaluate participants follow the recommendation of the 1st European Federation of Clinical Chemistry and Laboratory Medicine (EFLM) Strategic Conference (Milan 2014) [17]. 

The participating laboratories have improved their performance over the years, as is shown by increased compliance with the EPA (Figure 1) and a decrease in deviations from target value. The use of the BV-derived criterion to evaluate the results probably contributed to this improvement.

Despite criticism of the use of BV as a performance specification because it is too strict [17], this study shows that laboratories can achieve them for many biological magnitudes.

The decrease in dispersion of methods, the cessation of obsolete methods (enzymes) and the use of chemiluminescence in immunoassays (Figure 2) are contributing factors in improved performance.

It is difficult to compare SEQC^ML^ EQA programmes with other programmes from different organisations because the reports are only accessible to participants. This study therefore only presents data from programmes that publish their results in international journals or on open access websites. Other difficulties lie in the differences in the control materials distributed, the criteria for forming homogeneous groups, the calculations performed and in the quality specifications applied to assess the results.

The lower PD% of the SEQC^ML^ compared to ProBioQual (Table 2) are probably due to the fact that the Spanish programme uses BV as a specification, while the French programme uses the more permissive current method performance.

However, the discrepancies observed in the three programmes that use commutable controls with values assigned by certified reference methods (Table 3) cannot be explained in light of the data available in this study.

When the EPAs are based on state of the art, it has been found that most programmes do not define the ‘state of the art’ as the best *possible performance of* the methods used by the participants, as recommended at the 1st EFLM Strategic Conference in Milan [18], but as the *current performance* of the methods. This is a further difficulty in achieving harmonisation.

The data shown in this study reveal that, despite the fact that performance is continuously improving within each programme and efforts are being made for harmonisation, the results obtained under different programmes for some biological magnitudes are still not harmonised.

Harmonisation could be achieved through the efforts of all parties involved in the activity of the medical laboratory: international organisations that promote standards and guidelines, scientific societies that disseminate their data, the *in vitro* diagnostic industry that puts standardised methods and calibrated analytical systems on the market with documented traceability standards, and healthcare laboratories that use the best methods with rigour and protocolisation.

Since 1998 the international community has been making efforts to achieve harmonisation, as reflected in the following documentation:

– European Directive for the In Vitro Diagnostic Industry (1998), requiring suppliers to ensure traceability at the highest reference level, wherever possible.
– ISO 17511 (2003): In vitro diagnostic medical devices-Measurement of quantities biological samples – Metrological traceability of values assigned to calibration and control materials. This standard was published following the same requirements as the European directive [19]
– JCTLM (2002): established by several international bodies that keep a database of reference materials, analytical procedures and reference laboratories as per ISO 17511 [20]
– ICHCLR (2013): this consortium has a global portal where updated information on the status of standardisation/harmonisation of biological quantities is collected and protocols are developed to establish harmonisation of those biological quantities for which no reference materials and methods exist [21]
– The new ISO/NP 21151 standard (under development), defining protocols to be followed to ensure harmonisation of quantities without reference materials or methods [19]

EQA programmes play an important role in monitoring the harmonisation of medical laboratory performance, but the use of commutable controls with certified reference values should be encouraged wherever possible and the recommended analytical performance specifications applied.

In conclusions, the laboratories participating in the SEQC^ML^ EQA programmes have significantly improved their performance over the 30 years. The specifications derived from biological variation are achievable by many laboratories in our country. For biological quantities with no known BV data, the current state of the art is used, rather than the best possible. Organisers of EQA programmes should also join the harmonisation effort by making information on their results available to other interested professionals and by facilitating comparisons between organisations.

## Supplementary Material

Supplementary Material Details
