# Supplementary material for: Spanish society of laboratory medicine external quality assurance programmes: evolution of the analytical performance of clinical laboratories over 30 years and comparison with other programmes
Source: Adv Lab Med. 2020 May 19;1(2):20200019. doi: 10.1515/almed-2020-0019 (PMC10158744; doi:10.1515/almed-2020-0019)

Supplementary Figure 1. Report for the individual laboratory for category 4 programmes. Years 2003 and 2019


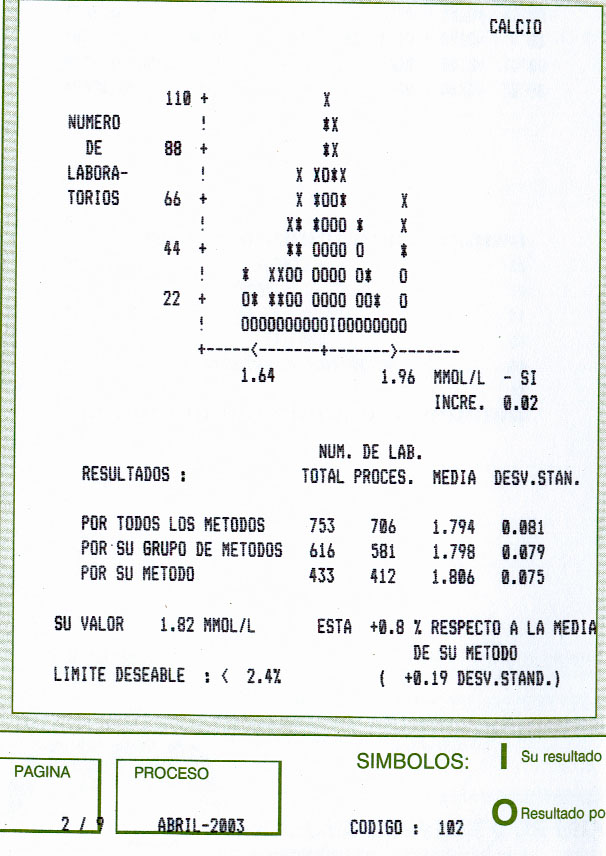

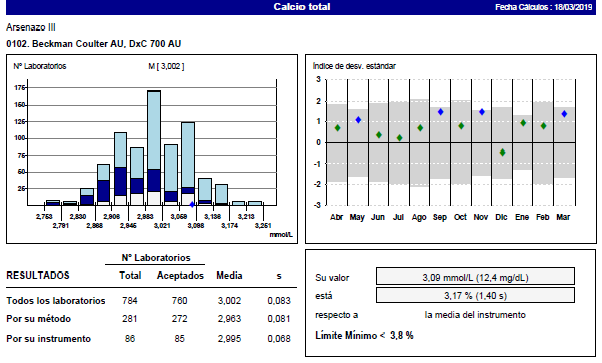


Supplementary Figure 2. Report for the individual laboratory for category 1 programmes.


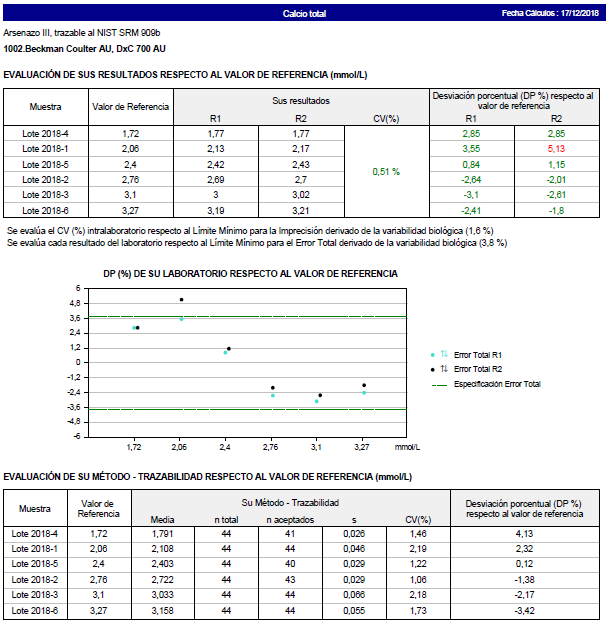


Supplementary Figure 3. Evolution of the 90th percentile of the PD% with respect to the homogeneous group (analytical ET) in the hormone programme


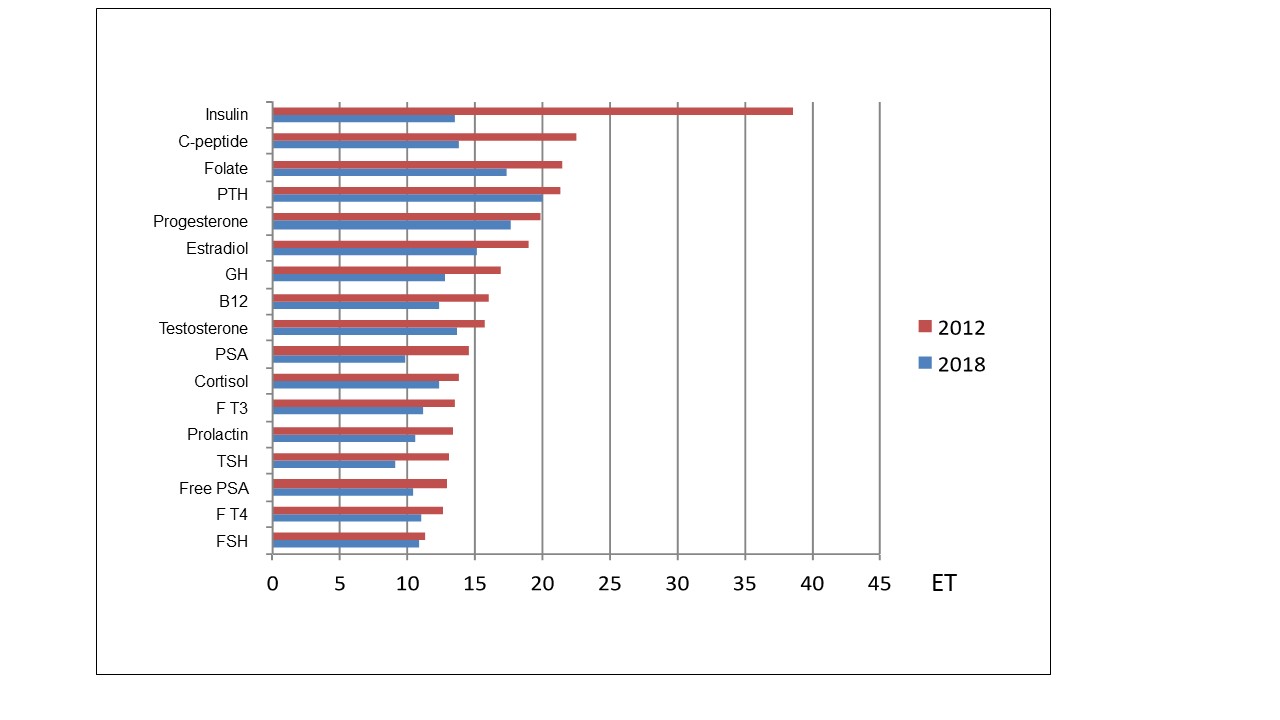

Supplement: Supplementary file 1 — Supplementary Material Details [file j_almed-2020-0019_suppl.docx]
